# T99. LONG-TERM CANNABIS USE ASSOCIATED WITH ALTERED FUNCTIONING DURING VERBAL LEARNING

**DOI:** 10.1093/schbul/sby016.375

**Published:** 2018-04-01

**Authors:** Grace Blest-Hopley, Aisling O’Neill, Robin Wilson, Vincent Giampietro, Sagnik Bhattacharyya

**Affiliations:** King’s College London, Institute of Psychiatry, Psychology, & Neuroscience

## Abstract

**Background:**

Long-term use of cannabis has long been associated with changes in cognition, including memory and learning, particularly verbal learning in man. However, evidence regarding the neurobiological underpinnings of impairments in memory following long-term cannabis use has not been consistent. Furthermore, to our knowledge none of the studies published to date have specifically investigated whether brain function differed between cannabis users and non-users while learning new information as estimated over repeated trials. Therefore, we aimed to investigate this.

**Methods:**

Twenty-one predominantly cannabis users (CU) who started using cannabis during adolescence and 21 healthy non-using controls (NU), completed a block design verbal paired associates learning task whilst undergoing functional Magnetic Resonance Imaging. The task required participants to learn and recall a set of word-pairs over 4 repeated trials. We examined the interaction between repetition and group (CU vs NU) on brain activation during encoding and recall condition using non-parametric repeated measures analysis of variance.

**Results:**

There was no significant difference in total recall score between CU and NU. However, there was a significant effect of repetition (p<0.001) on recall score, suggesting that there was a significant improvement in recall score over repeated trials across the two groups of participants. Furthermore, there was a significant interaction between repetition and group on recall score such that the change in recall score over repeated trials significantly differed (p =0.032) between the CU and NU groups. This was associated with a significant interaction (p =0.009) between group and repetition on activation in the midbrain bilaterally, extending to the, parahippocampus, caudate and cingulate gyrus during the encoding condition. There was greater engagement of these regions in CU than in NU over repeated encoding trials.

**Discussion:**

These results suggest that verbal learning is slower and more effortful requiring greater engagement of critical brain areas involved in learning in cannabis users compared to non-users.

